# The journey of the patients with dementia in the Peruvian health system

**DOI:** 10.1002/alz70860_103153

**Published:** 2025-12-23

**Authors:** Maria Lazo Porras, Francisco Tateishi Serruto, Christopher R. Butler, María Sofía Cuba, Daniela Rossini Vilchez, Silvana Perez León Quinoso, Miriam Lucar Flores, Jaime Miranda, Antonio Bernabe, Francisco Diez Canseco, Graham Moore, Filipa Landeiro, Maria Kathia Cardenas, Carlos Vera Tudela, Rafael A Calvo, William Whiteley, Jemma Hawkins

**Affiliations:** ^1^ CRONICAS, Universidad Peruana Cayetano Heredia, Lima, Lima, Peru; ^2^ Cronicas, Universidad Peruana Cayetano Heredia, LIma, Lima, Peru; ^3^ Department of Brain Sciences, Imperial College London, UK, London, London, United Kingdom; ^4^ Universidad Peruana Cayetano Heredia, Lima, Lima, Peru; ^5^ CRONICAS Centro de excelencia en enfermedades crónicas ‐ Universidad Peruana Cayetano Heredia, Lima, Lima, Peru; ^6^ CRONICAS, Universidad Peruana Cayetano Heredia, Perú, Lima, Lima, Peru; ^7^ CRONICAS, Universidad Peruana Cayetano Heredia, Lima, Peru; ^8^ Cardiff University, Cardiff, Wales, United Kingdom; ^9^ University of Oxford, Oxford, Oxfordshire, United Kingdom; ^10^ Dyson School of Design Engineering, Imperial College London, London, United Kingdom; ^11^ University of Edinburgh, Edinburgh, United Kingdom; ^12^ Cardiff University, Cardiff, United Kingdom

## Abstract

**Background:**

Dementia is a high burden condition in many low‐ and middle‐ income countries (LMIC). However, data are limited in terms of the patients' journey to receive a dementia diagnosis and treatment. There is also a lack of guidance on how to conduct this kind of research. This is the first study of its kind in Peru, providing a patient‐centered approach to understand the patient journey of people with dementia (PWD) and their carers. Understanding this journey is essential for improving healthcare practices that address the unique challenges faced in this context

**Method:**

This qualitative study used in‐depth interviews to map the patient journey of PWD. Participants included PWD, carers, and healthcare workers. A draft of the journey map was shared with a sub‐sample of participants for validation, involving individual and group discussions at each site. Participants reflected on their experiences, identifying missing steps, emotions, touch points, timelines, and actors involved.

**Result:**

We interviewed 39 participants: 4 PWD (2 female, 2 male), 18 carers (15 female, 3 male), and 17 healthcare workers (12 female, 5 male). Mapping the patient journey revealed that PWD and carers navigate more steps than healthcare professionals perceive. They often make logistical decisions, engaging with multiple health systems before obtaining a diagnosis and treatment. The validation process highlighted missing details in the initial map. Participants offered valuable feedback, enriching the map to better reflect the fragmented pathways to dementia care in Peru. Additionally, these sessions provided a supportive environment for carers and healthcare workers to share experiences, fostering a sense of community and engagement with the research process.

**Conclusion:**

Mapping the experiences of PWD and carers revealed longer, more complex journeys than previously recognised by healthcare professionals. The validation process further enriched the findings, providing key insights into navigating the Peruvian health system for dementia care and highlighting the importance of patient‐centred approaches in improving healthcare delivery.

